# Chemo-Nonresponsive Telangiectatic Osteosarcoma With Oligometastatic Recurrence Managed With Multimodality Therapy Including Stereotactic Ablative Body Radiotherapy (SABR): A Case Report With a Pathologic Treatment Effect

**DOI:** 10.7759/cureus.106242

**Published:** 2026-03-31

**Authors:** Wayne S Orr, Srinivasan Vijayakumar, Youssef Al Hmada, Paul Mobit, Anderson B Collier, Jennifer Barr, Pierre E De Delva, Derek B Davis, Claus Chunli Yang, Roselin Nittala

**Affiliations:** 1 SOM-Surgery-Transplant, University of Mississippi Medical Center, Jackson, USA; 2 Radiology, Ochsner Clinic Foundation and Ochsner Health, New Orleans, USA; 3 Radiation Oncology, Manipal Comprehensive Cancer Care Centre, Kasturba Medical College and Manipal Academy of Higher Education, Manipal, IND; 4 Cancer Care, Cancer Care Advisors and Consultants LLC, Ridgeland, USA; 5 Pathology, University of Mississippi Medical Center, Jackson, USA; 6 Radiation Oncology, University of Mississippi Medical Center, Jackson, USA; 7 Pediatric Hematology-Oncology, Nemours Children’s Health, Jacksonville, USA; 8 Orthopedic Oncology, University of Mississippi Medical Center, Jackson, USA; 9 Surgery, University of Mississippi Medical Center, Jackson, USA; 10 Pediatric Oncology, University of Mississippi Medical Center, Jackson, USA

**Keywords:** chemoresistant tumor cells, innovation in medicine, metastatic osteosarcoma, osteosarcoma research, precision cancer medicine, precision immuno-oncology, precision medicine oncology, radiation and clinical oncology, radiobiotherapy, stereotactic ablative body radiotherapy

## Abstract

Osteosarcoma is an aggressive primary bone malignancy that predominantly affects children, adolescents, and young adults. Outcomes remain poor for patients with high-risk features such as limited histopathologic response to neoadjuvant chemotherapy and early metastatic relapse. Other poor prognostic factors are a large tumor size, local recurrence, and pulmonary metastases.

We report a young adult male with a large telangiectatic osteosarcoma of the ulna with minimal necrosis after neoadjuvant methotrexate, doxorubicin (Adriamycin), and cisplatin (MAP), who declined further systemic chemotherapy after definitive surgery. He subsequently developed early pulmonary metastases and later bilateral adrenal metastases, managed with repeated metastasis-directed interventions guided by multidisciplinary tumor board deliberations and patient-centered decision-making.

A notable feature of this case is the use of modern radiotherapy, including preoperative stereotactic body radiotherapy (SBRT) for a locally recurrent right adrenal bed lesion, followed by surgical resection and intraoperative radiotherapy. The SBRT used high fraction sizes of 8 Gy per fraction and was described as stereotactic ablative body radiotherapy (SABR). Histopathologic evaluation of the resected specimen demonstrated an extensive treatment effect with no viable tumor cells.

Over nearly a decade of follow-up, the patient has maintained prolonged survival and good quality of life despite multiple recurrences and an initial lack of chemosensitivity. This case highlights how integrated, patient-centered multimodality care can be applied in complex osteosarcoma and provides pathologic evidence consistent with a marked SABR treatment effect in an adrenal tumor bed recurrence. These observations are hypothesis-generating and support further study in selected osteosarcoma settings.

## Introduction

Osteosarcoma is an uncommon primary malignancy of bone that disproportionately affects children, adolescents, and young adults [1,2]. Standard management integrates multi-agent chemotherapy and definitive surgery [1-3], and response to neoadjuvant chemotherapy, commonly measured by percentage tumor necrosis, remains one of the most informative prognostic indicators [2-5]. In contemporary practice, patients with localized disease who respond well to neoadjuvant therapy and undergo complete resection can achieve long-term survival [2-5]. However, outcomes remain poor in patients with high-risk features such as large tumor burden, early relapse, and limited pathologic response to neoadjuvant chemotherapy [2-6].

Despite these challenges, progress in osteosarcoma care increasingly depends on integrating multiple advances rather than reliance on a single modality [2,5]. Improvements in cross-sectional and functional imaging for earlier detection of limited-volume metastatic disease, refinements in thoracic and abdominal oncologic surgical techniques, and highly conformal radiotherapy (RT) techniques such as stereotactic body radiotherapy (SBRT), stereotactic ablative body radiotherapy (SABR) and intraoperative radiotherapy (IORT) have expanded the therapeutic options for patients with oligometastatic or locally recurrent disease [7-9]. Emerging evidence suggests that high-dose-per-fraction RT may induce immunogenic cell death and modulation of the tumor microenvironment, potentially contributing to local control beyond direct cytotoxic effects [10,11]. The concept of radio-biotherapy, the integration of targeted agents with RT to exploit mechanistic synergy, has been proposed as a framework for optimizing treatment combinations [12,13]. Biological concepts supporting SABR include indirect cell death and microenvironmental effects, and ongoing work seeks strategies to mitigate radio resistance [10-12].

We present the case of a young adult with telangiectatic osteosarcoma (TOS) of the ulna who exhibited minimal necrosis following neoadjuvant methotrexate, doxorubicin, and cisplatin (MAP) chemotherapy, declined further systemic chemotherapy after amputation, developed early pulmonary metastases, and later developed adrenal metastases. Over nearly a decade of follow-up, the patient achieved prolonged survival and sustained quality of life through repeated metastasis-directed interventions and longitudinal multidisciplinary coordination. Multidisciplinary tumor boards (MDTBs) play a central role in coordinating complex, multimodality care and incorporating patient preferences into treatment planning [14]. For instance, in this case report, patient's desire not to undergo chemotherapy was respected. Yet, he achieved prolonged survival through a series of metastasis-directed interventions guided by MDTB deliberations and patient-centered decision-making, including the use of precision medicine concepts [14-16]. Cabozantinib [17-43], a non-specific tyrosine kinase inhibitor (TKI), was used relatively soon after its FDA approval, potentially helping him. 

A distinctive component of this case is the demonstration of marked histopathologic treatment effect following preoperative SABR to a locally recurrent abdominal tumor bed lesion, followed by resection and IORT, with pathology demonstrating no viable tumor cells in the resected specimen, an unusual finding in osteosarcoma. Other aspects of this case that need to be expanded briefly are the telangiectatic histopathology, oligometastatic nature of the recurrences and their implications. The standard doses, complications and dose modifications of MAP chemotherapy and cabozantinib will also be mentioned.

TOS is a rare, high-grade osteosarcoma variant that often behaves aggressively and can present with rapid progression and a high risk of pathologic fracture because of its markedly lytic, blood-filled/cystic appearance. Clinically, outcomes are primarily driven by stage at diagnosis (especially the presence of metastases) and the ability to achieve complete local control with multimodality therapy. Population-based analyses suggest that when adjusted for stage and treatment, overall survival can be comparable to conventional osteosarcoma rather than intrinsically worse [44-46].

Standard neoadjuvant chemotherapy for newly diagnosed high‑grade osteosarcoma is most commonly the MAP regimen (high‑dose methotrexate, doxorubicin (Adriamycin), and cisplatin), administered preoperatively to treat micrometastatic disease, facilitate limb‑sparing resection when feasible, and enable prognostic assessment of histologic response (percent necrosis) at surgery. Commonly used cooperative‑group MAP schedules employ high‑dose methotrexate 12 g/m^2^ per dose (typically given as two doses per cycle with pharmacokinetically guided leucovorin rescue), doxorubicin 75 mg/m^2^ per cycle, and cisplatin 120 mg/m^2^ per cycle (often fractionated over two days), with postoperative continuation when tolerated. Dose modifications are individualized but generally include delaying/holding high‑dose methotrexate for renal dysfunction, third‑space fluid collections, or delayed drug clearance with escalation of supportive measures (hydration/urine alkalinization) and leucovorin rescue based on measured methotrexate levels, reducing/holding cisplatin for nephrotoxicity and ototoxicity, and limiting anthracycline exposure in the setting of reduced cardiac function and cumulative dose thresholds [1,5,47-49].

Cabozantinib is an oral multikinase inhibitor (including MET and VEGFR2) used in relapsed/refractory osteosarcoma, with the CABONE phase 2 trial using 60 mg once daily in adults (and BSA-based dosing in younger patients), and real‑world series similarly initiating most adults at 60 mg daily. Common toxicities include diarrhea, fatigue, anorexia/weight loss, palmar-plantar erythrodysesthesia, hypertension, mucositis/stomatitis, and laboratory abnormalities (e.g., transaminase elevation), and these are typically managed with supportive care plus treatment interruption and stepwise dose reduction. For CABOMETYX tablets, a commonly used dose‑reduction sequence is 60→40→20 mg once daily (or 40→20 mg once daily when starting at 40 mg), with drug holding for clinically significant (e.g., intolerable grade 2 or grade ≥3) adverse events until improvement (often to grade ≤1) and then resumption at a reduced dose; in practice, diarrhea and hand-foot syndrome are among the most frequent reasons for dose interruption/reduction [17-19,25].

In 1995, Hellman and Weichselbaum [50] introduced the term “oligometastases,” proposing a clinically meaningful intermediate state between localized disease and widely disseminated metastases in which a limited number of metastatic deposits may be amenable to curative-intent local therapy. In modern practice, oligometastatic disease is viewed as a spectrum and dynamic state, increasingly detected with improved imaging and potentially addressable with metastasis-directed therapies such as surgery and SABR/SBRT, often integrated with effective systemic therapy. SBRT/SABR refers to delivery of highly conformal, image-guided external-beam radiation with steep dose gradients using a small number of fractions and a high dose per fraction to ablate discrete targets; because toxicity risk is strongly tied to geometric uncertainty, safe implementation typically relies on rigorous target localization, motion management (when relevant), and quality assurance [51-54]. Recent reviews and meta-analyses across solid tumors support that, in appropriately selected patients, adding metastasis-directed local therapy (including SBRT/SABR) can improve progression-related outcomes and, in some settings, overall survival [55-58], while emphasizing the ongoing need for refined patient selection and standardized definitions. In parallel, emerging concepts in radiation medicine emphasize biologically informed integration of RT with systemic agents [12,13], providing a conceptual framework for combining metastasis-directed RT with targeted therapies and other modern systemic approaches [10,12,13,50-58].

This report aims to highlight (1) how multidisciplinary, patient-centered precision cancer care can be operationalized in complex osteosarcoma management [14,15], (2) how modern radiotherapeutic concepts and technologies may warrant renewed consideration in carefully selected osteosarcoma scenarios, particularly when combined with metastasis-directed surgery, close surveillance [1-13] and use of a targeted agent such as cabozantinib with modern RT using precision medicine principles [12,13,15], and (3) finally, how aggressive interventions in the oligometastatic disease state can help achieve long-term disease control.

This article was presented as an invited Podium Lecture at the 5th Annual NEAUX CANCER CONFERENCE of the Cancer Advocacy Group of Louisiana (CAGLA), New Orleans, LA, March 18-20, 2026.

## Case presentation

**Figure 1:**
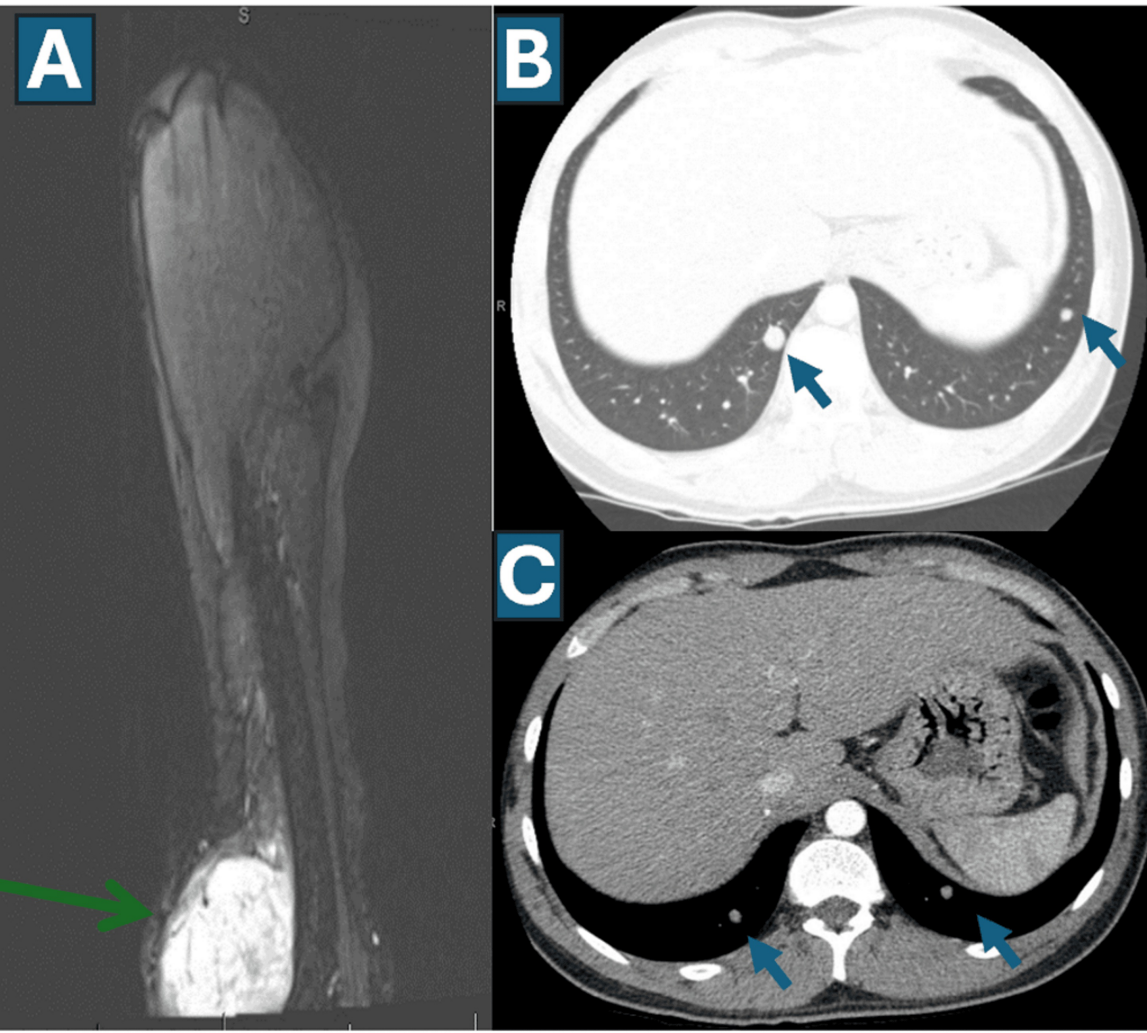
Sequential representative images from the time of diagnosis (A), followed by when lung metastases were first identified (B and C) A. MRI of the left arm showing the mass (blue arrow) arising from the ulna (at initial presentation). B. CT chest with intravenous contrast showing bilateral pulmonary metastases (blue arrows showing two representative lesions). C. Thin slices (1.5 mm) further confirming the findings as in B (blue arrows showing two representative lesions).

**Figure 2:**
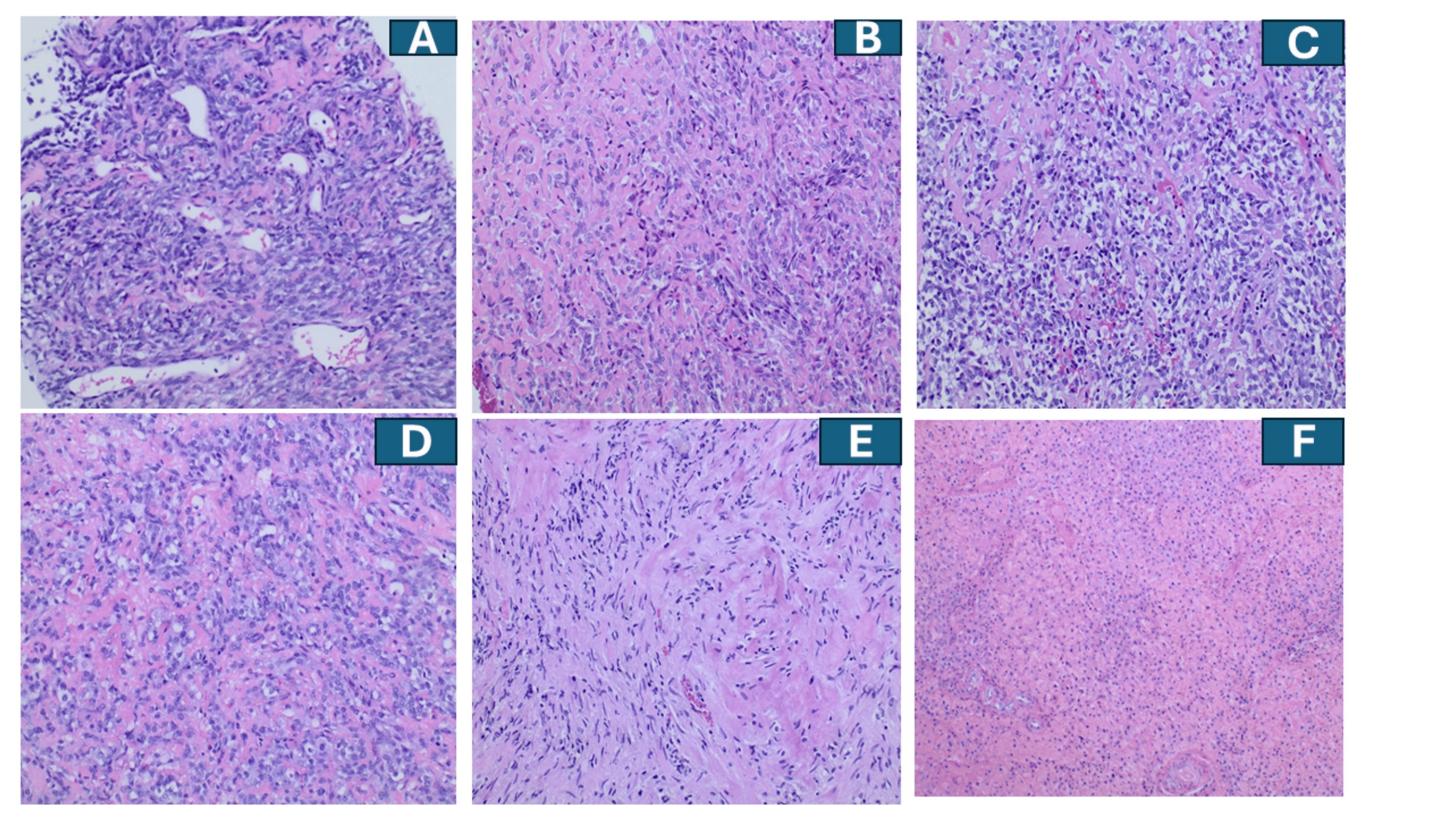
Sequential representative histopathology images from biopsy (A) followed by surgical resection, (B) lung and adrenal metastases (C and D), and finally E and F showing SABR-induced treatment effects A demonstrates solid sheets of tumor cells with multiple vessels and osteoid formation, diagnostic of osteosarcoma (telangiectatic variant). B shows histopathology from the above-elbow amputation specimen after neoadjuvant MAP chemotherapy (11/2016), showing sheets of malignant cells producing prominent osteoid with limited treatment-related necrosis, consistent with poor histopathologic response. C is a representative lung metastasis histopathology, showing epithelioid cells with osteoid production, diagnostic of metastatic epithelioid osteosarcoma. D is a representative histopathological image of adrenal metastasis. E shows a microscopic picture after SABR representative of a remarkable response to radiotherapy; the figure shows epithelioid cells with extensive hemorrhage and ischemic changes with no recognizable tumor cells. Findings from F are also consistent with findings from E, showing reactive fibroblastic proliferation, scattered inflammatory cells, and hyalinization consistent with SABR treatment effects. SABR: Stereotactic ablative body radiotherapy

A 24-year-old Hispanic male presented with a large swelling in the left elbow region and was diagnosed on 07/2016 with TOS of the left ulna (Figure 1A). Initial staging showed no evidence of metastatic disease. After MDTB discussion, he received neoadjuvant MAP chemotherapy (methotrexate, doxorubicin, and cisplatin) following standard protocols [5,47-49], followed by definitive surgery (above-elbow amputation on 11/2016). Pathology demonstrated limited treatment-related necrosis (Figures 2A, 2B), a poor prognostic indicator [2-6], and adjuvant systemic therapy was recommended; however, the patient declined further chemotherapy after informed discussion. Close surveillance was recommended.

Table 1 summarizes the most important events in the patient’s disease course and corresponding management decisions.

**Table 1:** Disease course from diagnosis in July 2016 to March 2026 showing key findings and interventions IORT: Intraoperative radiotherapy; SBRT: stereotactic body radiotherapy

Date	Key findings and interventions
07/2016	Diagnosis: Telangiectatic osteosarcoma of the left ulna (Figure 1A); staging workup negative for metastases.
08/2016	Neoadjuvant therapy: MAP chemotherapy.
11/2016	Definitive surgery: Above-elbow amputation; pathology showed minimal necrosis (Figures 2A, 2B). Adjuvant chemotherapy recommended; patient declined.
01/2017	Metastatic relapse: Surveillance CT chest demonstrated bilateral pulmonary metastases (Figures 1B, 1C). Systemic therapy recommended; patient declined.
03/2019	Disease progression: PET/CT showed worsening pulmonary metastatic disease (Figures 3A–3D).
04/2019	Surgery: Bilateral pulmonary metastasectomy (13 lesions total: seven right, six left) (Figure 2C; representative histopathology).
08/2020	New metastases: Bilateral adrenal metastases detected on imaging (Figures 4A–4C).
09/2020	Targeted therapy: Cabozantinib initiated; partial response noted by early 2022 (Figures 4D, 4E).
05/2021	Surgery: Bilateral adrenalectomy (Figure 2D; representative histopathology). Clinically disease-free postoperatively; continued cabozantinib with steroid replacement.
07/2022	Local recurrence: New ~3 cm lesion in the right adrenal bed consistent with recurrence (Figure 4F); no distant metastatic disease noted.
09/2022	Radiation: Preoperative SBRT to right adrenal bed recurrence: 40 Gy in five fractions over 10 days (Figure 5).
10/2022	Surgery + IORT: Resection of right adrenal bed recurrence (clinically R0) and IORT (10 Gy) to tumor bed (Appendix, Figures 5, 6). Pathology: no viable tumor cells (Figures 2E, 2F; compare with Figures 2A–2D).
02/2024	Treatment hold: Cabozantinib held due to diarrhea; imaging showed no evidence of disease.
08/2024–12/2025	Ongoing management: Indeterminate mild uptake/changes in adrenal beds discussed at tumor board; cabozantinib restarted and dose-reduced (30 mg daily) due to diarrhea.
03/2026	Suspected recurrence: PET/CT suggested likely local recurrence in the left adrenal bed; management under discussion.

**Figure 3:**
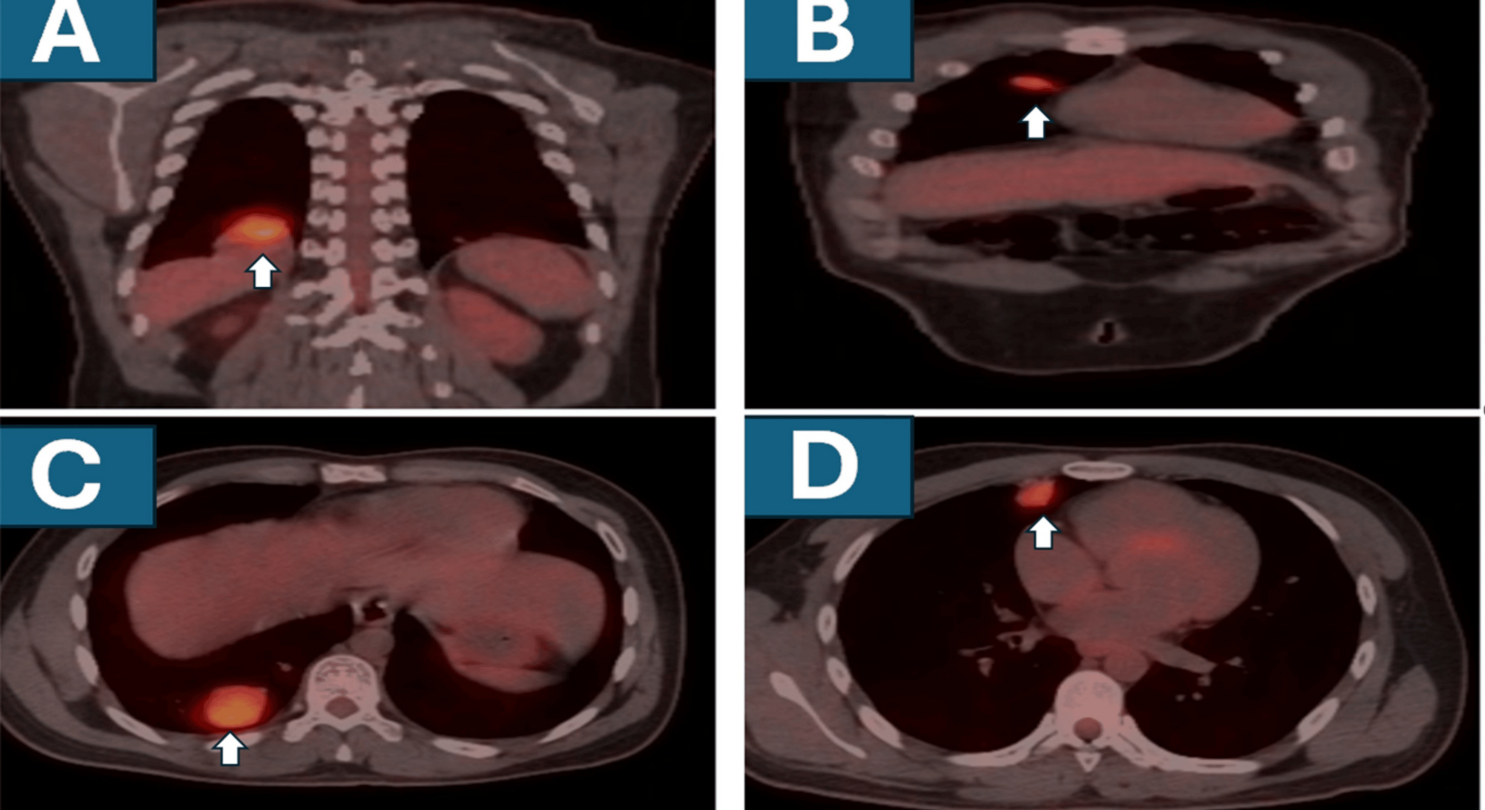
Representative PET/CT images showing progression of the pulmonary metastases A. PET/CT, coronal view showing increased metabolic activity in a pulmonary metastatic lesion on the right above the diaphragm (white arrow). B. Another coronal view showing another pulmonary metastasis with increased metabolic activity in the right lung at a higher level (white arrow). C. PET/CT fused axial image showing a pulmonary lesion with increased metabolic activity posteriorly in the right lung base (white arrow). D. Another axial image showing another pulmonary lesion with increased metabolic activity on the right, posterolateral to the sternum (white arrow).

In 12/2017, routine surveillance CT of the chest demonstrated bilateral pulmonary metastases (Figures 1B, 1C). Systemic therapy was again recommended; the patient declined, and he was followed with close imaging surveillance. By March 2019, PET/CT showed worsening pulmonary metastatic disease (Figures 3A-3D).

**Figure 4:**
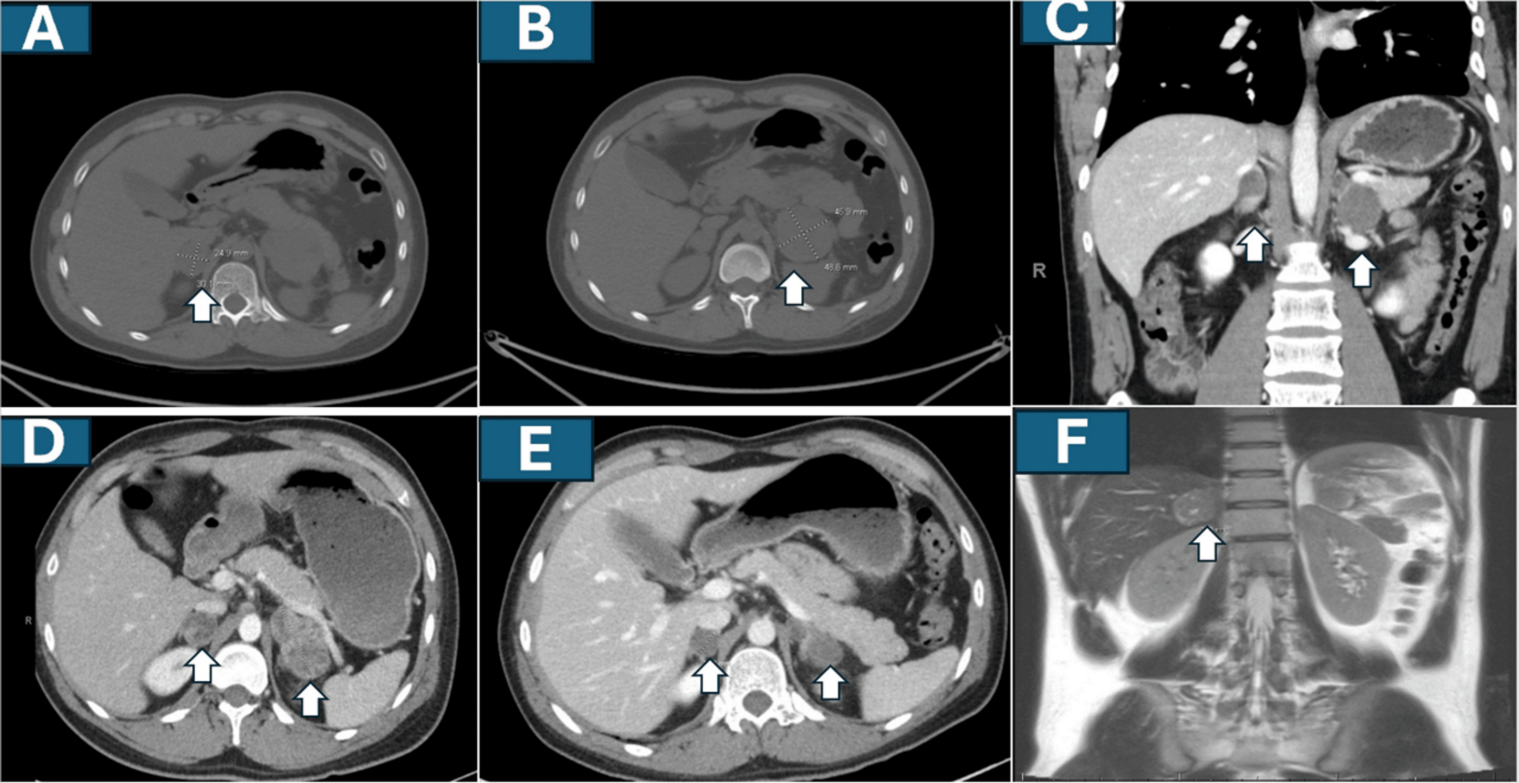
Sequential representative CT (A-E) and MRI (F) images showing patient's disease course after pulmonary metastasectomy A shows an axial CT slice with right adrenal metastasis (white arrow); B shows in another axial view left adrenal metastasis (white arrow); C is a coronal CT view showing bilateral adrenal metastases (white arrows). D and E show axial CT images showing partial response to cabozantinib leading to bilateral oligometastatectomy of adrenals (white arrows). MRI coronal view in F shows recurrence in the right adrenal tumor bed prior to SABR preoperative radiotherapy (white arrow). SABR: Stereotactic ablative body radiotherapy

He underwent bilateral thoracotomy with metastasectomy [16] in 04/2019 (13 lesions were removed in total; seven on the right and six on the left) (Figure 2C, a representative histopathology of lung metastasis). The patient again declined adjuvant chemotherapy. In August 2020, surveillance imaging identified bilateral adrenal metastases (Figures 4A-4C).

After multidisciplinary discussion and in alignment with the patient’s preference to avoid cytotoxic chemotherapy, cabozantinib [17-43] was initiated at 40 mg per day orally in September 2020. He achieved a partial response as shown in Figures 4D, 4E (compare with Figures 4A-4C). Given the oligometastatic pattern, MDTB favored another metastasectomy and he underwent bilateral adrenalectomy in 05/2021 (Figure 2D showing histopathology) and was maintained on steroid replacement therapy. Cabozantinib continued postoperatively.

Surveillance imaging in July 2022 demonstrated a new ~3 cm lesion in the right adrenal bed abutting the hepatic lobe (Figure 4F), without evidence of distant metastatic disease. The MDTB recommended an aggressive local approach given that this is the only site of demonstrable tumor recurrence with no other evidence of distant metastasis. He received preoperative SABR to the lesion (40 Gy in five fractions) (see the Appendix for the details) from the first week of 09/2022 to the middle of 09/2022 over 10 days, followed by surgical resection in the third week of 10/2022 with clinically complete (R0) resection. Given the history of local recurrence in a postoperative bed, IORT was delivered to the resection bed (10 Gy) (see the Appendix).

Grossly, the mass was described intraoperatively as largely liquefactive/necrotic. Histopathologic evaluation of the resected specimen demonstrated extensive treatment effect with no viable tumor cells identified (Figures 2E-2F; also compare with Figures 2A-2D of the primary tumor and other metastatic lesions histopathological appearances which in contrast show abundant viable tumor cells).

He recovered without complications and continued with surveillance. In February 2024, cabozantinib was temporarily discontinued because of diarrhea; imaging at that time showed no evidence of disease. Subsequent surveillance noted persistent mild/indeterminate findings in the adrenal beds that were reviewed in a multidisciplinary conference, with uncertainty between postoperative change and recurrence; cabozantinib was restarted in August 2024 and dose-reduced to 30 mg per day due to diarrhea. In early March 2026, PET/CT suggested a likely local recurrence in the left adrenal bed, and management options are under discussion. There appears to be no recurrence in the right adrenal tumor bed or any evidence of other distant metastases. Throughout most of the disease course, the patient maintained good functional status and quality of life, including continued employment, with diarrhea as the main treatment-limiting symptom.

## Discussion

This case illustrates several important principles in the management of high-risk osteosarcoma: (1) the prognostic significance of limited histopathologic response to neoadjuvant chemotherapy, (2) the role of metastasis-directed therapy in oligometastatic disease, (3) the integration of targeted therapy with multimodality local treatment, (4) the potential role of high-dose-per-fraction RT in osteosarcoma, and (5) the importance of MDTBs and patient-centered decision-making in complex oncologic care.

Limited histopathologic response to neoadjuvant chemotherapy

Histopathologic response to neoadjuvant chemotherapy, commonly measured by percentage tumor necrosis, is one of the most informative prognostic indicators in osteosarcoma [2-6]. Patients with ≥90% necrosis (good responders) have significantly better outcomes than those with <90% necrosis (poor responders) [2-6]. In this case, the patient demonstrated limited necrosis after neoadjuvant MAP chemotherapy, placing him in the poor-responder category and at high risk for relapse. The early development of pulmonary metastases approximately four months after surgery was consistent with this high-risk profile.

Recent advances in digital pathology and artificial intelligence have enabled more precise quantification of viable tumor cell density after neoadjuvant chemotherapy, with potential to refine prognostic stratification [6]. However, the fundamental principle that histopathologic response predicts outcome remains central to osteosarcoma management [1-6].

Metastasis-directed therapy in oligometastatic disease

The concept of oligometastatic disease, a state of limited metastatic burden that may be amenable to metastasis-directed therapy, has gained increasing recognition across multiple tumor types. In osteosarcoma, pulmonary metastasectomy has been a standard component of management for decades, with long-term survival reported in selected patients who achieve complete resection [1-5,16]. This case extends that principle to extrapulmonary oligometastatic disease, with bilateral adrenalectomies performed for isolated adrenal metastases and subsequent resection of a locally recurrent adrenal bed lesion.

The decision to pursue aggressive metastasis-directed therapy in this case was guided by several factors: (1) limited number and sites of metastatic disease, (2) absence of rapidly progressive systemic disease, (3) patient’s refusal of systemic chemotherapy, and (4) availability of targeted therapy (cabozantinib) to address potential micrometastatic disease. These decisions were made through MDTB deliberations that incorporated patient preferences and values into treatment planning [14,15].

Integration of targeted therapy with multimodality local treatment

Cabozantinib is an orally bioavailable, small-molecule multikinase TKI that inhibits several receptor tyrosine kinases implicated in oncogenesis and tumor microenvironment biology, most notably MET (HGFR) and VEGFR2, with additional activity against RET, KIT, AXL, and FLT3 [17-33]. The drug was initially approved by the FDA for medullary thyroid cancer in 2012 [24,25] and subsequently for advanced renal cell carcinoma (RCC) in 2016 [26,27].

Preclinical studies have demonstrated that cabozantinib affects osteosarcoma growth through direct effects on tumor cells and modifications in the bone microenvironment [23,28,30,31]. The CABONE trial, a multicenter, single-arm, phase 2 trial, evaluated cabozantinib in patients with advanced Ewing sarcoma or osteosarcoma [17]. While the trial did not meet its primary endpoint of progression-free survival at 12 weeks, a subset of patients demonstrated prolonged disease control, and the drug was generally well tolerated [17]. Real-world data have similarly shown that some patients with advanced osteosarcoma or Ewing sarcoma achieve prolonged disease control with cabozantinib [17-20].

In this case, cabozantinib was initiated after the development of bilateral adrenal metastases, with partial response observed on imaging. The drug was continued through bilateral adrenalectomies and subsequent local recurrence, and the patient remains on cabozantinib at the time of this report. While it is not possible to definitively attribute the patient’s prolonged survival to cabozantinib, the drug may have contributed to control of micrometastatic disease and facilitated the success of metastasis-directed interventions.

An ongoing phase 3 trial (NCT05691478) is evaluating the addition of cabozantinib to standard chemotherapy in patients with newly diagnosed osteosarcoma [38]. Results from this trial may provide definitive evidence regarding the role of cabozantinib in osteosarcoma management.

High-dose-per-fraction radiotherapy in osteosarcoma

Osteosarcoma has historically been considered a radioresistant tumor, and RT has traditionally been reserved for unresectable or incompletely resected disease [7]. However, emerging evidence suggests that high-dose-per-fraction RT, such as SABR, may achieve meaningful local control in selected osteosarcoma settings [8,9]. The biologic mechanisms underlying the efficacy of high-dose-per-fraction RT may extend beyond direct cytotoxic effects to include immunogenic cell death and modulation of the tumor microenvironment [10-12].

In this case, preoperative SBRT (40 Gy in 5 fractions, 8 Gy per fraction) was delivered to a locally recurrent adrenal bed lesion, followed by surgical resection and IORT (10 Gy). Histopathologic evaluation of the resected specimen demonstrated extensive treatment effect with no viable tumor cells (Figures 2E, 2F). While it is not possible to definitively attribute this treatment effect to SABR alone (as opposed to the combination of SABR and cabozantinib), the findings are consistent with a marked SABR treatment effect and support further study of high-dose-per-fraction RT in osteosarcoma.

The decision to proceed with surgery shortly after completion of SBRT (approximately five weeks) rather than waiting for a conventional 6-8-week interval was based on concern that prolonged delay might lead to fibrosis that would complicate surgical resection. This approach is consistent with emerging data suggesting that early surgery after SBRT may be feasible and safe in selected settings.

Cabozantinib and radiotherapy: safety, potential synergy, and clinical experience

The concurrent or sequential use of cabozantinib with RT has been explored in multiple tumor types, most extensively in metastatic RCC. Available clinical data suggest that the combination is generally safe when RT is delivered to limited volumes using modern conformal techniques, although caution is warranted in specific anatomic settings and with large-field RT, as discussed later.

Clinical experience with cabozantinib in metastatic RCC

The largest body of clinical experience with cabozantinib and RT comes from metastatic RCC [31]. A real-world study from the International Metastatic Renal Cell Carcinoma Database Consortium (IMDC) analyzed 127 patients with metastatic RCC treated with cabozantinib and RT [31]. Of these, 53% received concurrent RT (defined as RT delivered while on cabozantinib), and 6.3% experienced grade ≥3 adverse events [31]. The most common RT sites were bone (58%) and brain (28%), with most patients receiving palliative or stereotactic RT to limited volumes [31]. No consistent signal of enhanced acute radiation toxicity was observed with concurrent administration [31].

The CABOREAL Early Access Program, a prospective registry study of cabozantinib in metastatic RCC, similarly reported that concurrent RT or bone-targeted agents did not result in excess toxicity [32]. These data support the general safety of concurrent cabozantinib and RT in metastatic RCC, particularly when RT is delivered to limited volumes using modern conformal techniques.

FDA approvals and expanding indications

Beyond RCC, cabozantinib has received FDA approval for multiple indications. The FDA approved cabozantinib for previously treated hepatocellular carcinoma on January 14, 2019 [33], based on the CELESTIAL trial demonstrating improved overall survival compared to placebo. Most recently, on March 26, 2025, the FDA approved cabozantinib for previously treated advanced pancreatic and extra-pancreatic neuroendocrine tumors [37], based on the CABINET trial.

General lack of excessive complications with concurrent administration of cabozantinib and radiotherapy

Across available clinical series, no consistent signal of enhanced acute radiation toxicity has been observed when cabozantinib is administered concurrently with RT, particularly when RT is delivered to limited volumes using modern conformal techniques [31,32]. This distinguishes cabozantinib from earlier concerns raised with some VEGF-directed agents and suggests that routine short treatment interruptions may not be universally required, especially for palliative or stereotactic indications.

Nevertheless, rare but serious adverse events-such as delayed radiation recall reactions, soft-tissue necrosis, or fistula formation-have been reported in isolated cases, often occurring months after RT and typically in previously irradiated, high-risk anatomic regions [34,40-42].

Potential radiosensitization by cabozantinib: preclinical and in vitro evidence

A biologic rationale for radiosensitization by cabozantinib is supported by multiple preclinical studies. Cabozantinib inhibits MET‑ and AXL‑mediated DNA damage repair, reduces tumor hypoxia through anti‑angiogenic effects, and modulates tumor-stromal interactions. In vitro studies have demonstrated enhanced clonogenic cell kill when cabozantinib is combined with ionizing radiation [21,23,35].

More recently, mechanistic work in non-small cell lung cancer models demonstrated that cabozantinib enhanced radiation-induced cytotoxicity by promoting ferroptosis, mediated through suppression of the STAT3-MCL1-BECN1-SLC7A11 axis [35]. However, this effect is not universal across tumor types. In a murine breast cancer model, cabozantinib enhanced radiation response in vitro but did not improve tumor growth control in vivo, underscoring the context-dependent nature of potential synergy [35]. Robust clinical evidence demonstrating improved tumor control or survival with cabozantinib-RT combinations is currently lacking.

Importance of the radiotherapy technique and anatomic caution

Available data support the importance of RT field size and conformality when combining RT with cabozantinib. The safest clinical experiences have been reported with small-volume, highly conformal treatments, including SABR and focal palliative RT [31,32]. In contrast, large-field RT and irradiation of mucosal, tracheal, esophageal, or other hollow-organ structures may confer higher risk, particularly given cabozantinib’s association with impaired wound healing, hemorrhage, and fistula formation [41,42].

Accordingly, most experts recommend avoiding extensive elective fields, minimizing dose to critical mucosal surfaces, and exercising caution in re‑irradiation settings. These principles are especially relevant in head and neck, thoracic, and upper gastrointestinal RT.

Need for radiobiotherapy trials and future directions

Despite encouraging safety data and a biologic rationale, the combination of cabozantinib and RT remains insufficiently studied in prospective trials, including in osteosarcoma. The concept of “radiobiotherapy” has been proposed to guide carefully designed clinical trials integrating targeted agents with radiation to exploit mechanistic synergy while preserving safety [12,13,39,40]. Such trials are needed to define optimal sequencing, dose constraints, biomarkers of benefit, and tumor-specific indications for cabozantinib-RT combinations.

Summary: cabozantinib and radiotherapy

In summary, the largest available clinical experience, primarily in metastatic RCC, supports the general safety of concurrent cabozantinib and RT, particularly when RT is delivered to small, conformal target volumes [31,32]. Preclinical data suggest potential radiosensitization, but clinical efficacy signals remain unproven, and caution is warranted when irradiating large fields or mucosal structures [34,35,39-42]. Prospective radiobiotherapy trials are essential to determine whether biologic synergy can be translated into meaningful clinical benefit [12,13,31,32,40]. The importance of application of modern RT techniques and quality assurance protocols cannot be overemphasized [51-53].

Multidisciplinary tumor boards and patient-centered decision-making

This case highlights the central role of MDTBs in coordinating complex, multimodality care and incorporating patient preferences into treatment planning [14]. At multiple decision points, including the initial refusal of adjuvant chemotherapy, the development of pulmonary metastases, the development of adrenal metastases, and the local recurrence in the adrenal bed, MDTB deliberations guided treatment recommendations that balanced oncologic principles with patient values and preferences.

The patient’s consistent refusal of systemic chemotherapy was respected, and alternative strategies were developed that incorporated targeted therapy, metastasis-directed surgery, and high-dose-per-fraction RT. This patient-centered approach, facilitated by MDTB deliberations, enabled the patient to achieve prolonged survival and good quality of life despite high-risk disease features.

Limitations and cautions

This case report has several limitations. First, as a single case, it is not possible to draw definitive conclusions about the efficacy of any specific intervention. The patient’s prolonged survival may reflect favorable tumor biology, the cumulative effect of multiple metastasis-directed interventions, the contribution of cabozantinib, or some combination of these factors. Second, the histopathologic treatment effect observed in the resected adrenal bed specimen cannot be definitively attributed to SBRT alone, as the patient was also receiving cabozantinib and subsequently received IORT. Third, the patient’s refusal of systemic chemotherapy limits the ability to compare outcomes to standard-of-care treatment. Finally, the long-term durability of disease control remains uncertain, and continued surveillance is warranted.

Although this case illustrates many important potentials, this report is not meant to be a practice altering in its intentions. There is cautious optimism in the future success of the hypotheses leading to improved outcomes in osteosarcoma in the future, only future laboratory, translational, and clinical research can prove or disprove those outlooks.

## Conclusions

This case demonstrates that prolonged survival and good quality of life can be achieved in selected patients with high-risk, chemo-nonresponsive osteosarcoma through integrated, patient-centered multimodality care guided by MDTB deliberations. The histopathologic evidence of extensive treatment effect with no viable tumor cells in the resected adrenal bed specimen after preoperative SBRT is hypothesis-generating and supports further study of high-dose-per-fraction RT in osteosarcoma. The concurrent use of cabozantinib and RT was well tolerated in this case, consistent with emerging clinical experience in other tumor types. Prospective trials are needed to define the optimal integration of targeted therapy, high-dose-per-fraction RT, and metastasis-directed surgery in osteosarcoma management.

## Figures and Tables

**Figure 5:**
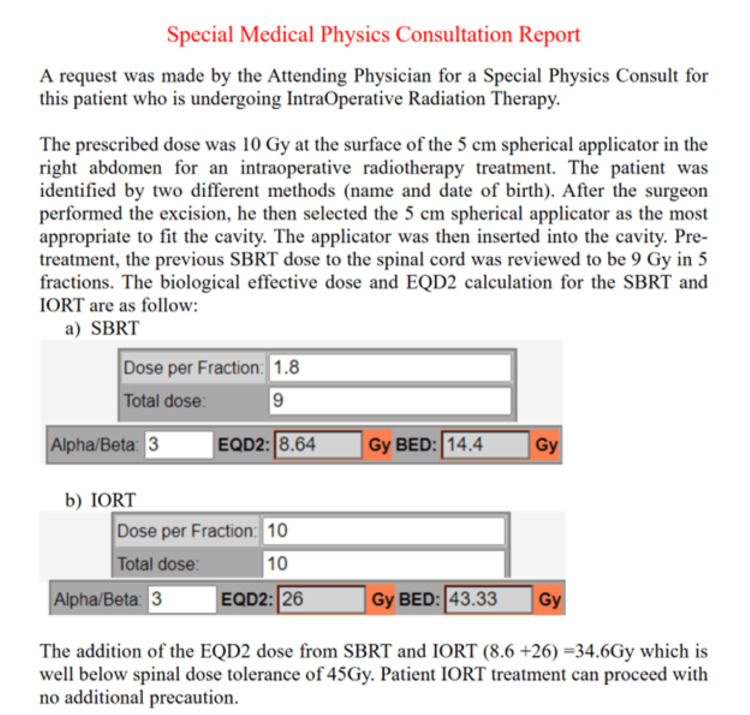
Technical details of IORT dose and cone sizes. The details of the placement and safety precautions are also described Medical physicists are integral interdisciplinary collaborative team members of the IORT team. They are present in the operation room at the time of IORT delivery to facilitate precise and accurate delivery of IORT as well as for the radiation safety purposes. They carefully monitor the dose delivery and collaborate closely with surgeons and radiation oncologists. IORT: Intraoperative radiotherapy

**Figure 6:**
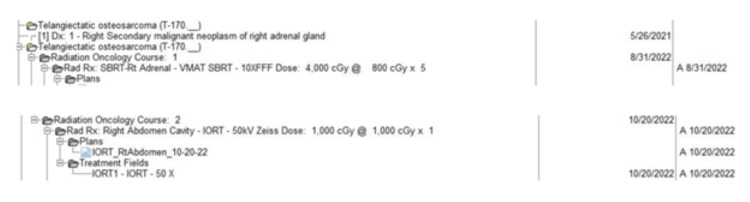
Technical details of stereotactic ablative body radiotherapy (SABR) and dose/fractionation scheme information for SABR and IORT The dose scheme for SABR is detailed. 4000 cGy was prescribed and delivered in 5 fractions. VMAT (volumetric modulated arc therapy) was used (upper half of the figure). For IORT, 1000 cGy was delivered in a single fraction using 50 KV X-rays to the right abdominal cavity (right adrenal tumor bed) IORT: Intraoperative radiotherapy

## Appendices

Technical details of stereotactic ablative body radiotherapy and intraoperative radiotherapy
